# Epigenomics dysregulation and therapeutics in neurodegeneration

**DOI:** 10.1002/alz.083398

**Published:** 2025-01-03

**Authors:** Anne‐Laurence Boutillier

**Affiliations:** ^1^ UMR7364 CNRS UNISTRA, Strasbourg, Alsace France

## Abstract

**Background:**

Aging is the main risk factor of cognitive neurodegenerative diseases such as Alzheimer’s disease, with epigenome alterations as a contributing factor, however, it is not clear which biological mechanisms are common or disease‐specific. Here we investigated epigenomic/genomic signatures in the hippocampus of mouse models of aging and of tauopathy, an AD‐related feature.

**Methods:**

Aging was modelled by comparing 18‐month‐ versus 3‐month‐old WT mice. Tauopathy was modelled using 12‐month‐old THY‐Tau22 mice. We used high resolution techniques interrogating the whole genome (e.g. high‐throughput sequencing such as Chromatin immunoprecipitation (ChIP) coupled to sequencing, combined with transcriptomics (RNA‐seq), on bulk tissues and at the cellular resolution (FANS‐sorted neuronal nuclei).

**Results:**

At the transcriptional level, expression of nearly all cholesterol biosynthesis genes was severely impaired in tauopathic mice compared to the model of physiological aging. At the epigenomic level, tauopathy induced hyperacetylation of histone H3 at lysine 27, a marker of active transcription, at neuronal enhancers mostly associated with excitatory neurons. The opposite was found in the aged hippocampus, suggesting that tauopathy is not an exacerbation of an epigenetic drift observed during the aging process. Furthermore, we show that cholesterogenic genes were highly expressed in WT and down‐regulated by tauopathy in CA hippocampal neurons, pointing to a specific vulnerability of these neurons to tauopathy. Lastly, a treatment of tau mice with CSP‐TTK21, a CBP/p300 HAT activator, restored expression of main cholesterol biosynthesis genes, and also unexpectedly counteracted hyperacetylation at neuronal enhancers. This treatment ultimately restored object memory.

**Conclusions:**

Our results indicate a link between decreased cholesterol biosynthesis pathway and hyperacetylation in tauopathy, likely through the availability of their common primary substrate, acetyl‐CoA. This work supports the use of molecules capable of activating the cholesterol/mevalonate pathway in the brain subjected to Tau‐related neurodegenerative cognitive diseases (including Alzheimer’s disease) and opens to the use of new epi‐drugs. We are now studying these regulations in response to environmental cues, such as social and physical enrichment, which are emerging as powerful modifiers of disease progression in many neurodegenerative diseases, although their ability to alter the epigenomic landscape of the brain remains poorly described.

